# *Z* boson production in bottom-quark fusion: a study of *b*-mass effects beyond leading order

**DOI:** 10.1140/epjc/s10052-018-6414-8

**Published:** 2018-11-14

**Authors:** Stefano Forte, Davide Napoletano, Maria Ubiali

**Affiliations:** 1 Tif Lab, Dipartimento di Fisica, Università di Milano and INFN, Sezione di Milano, Via Celoria 16, 20133, Milano Italy; 2 IPhT, CEA Saclay, CNRS UMR 3681, 91191 Gif-Sur-Yvette, France; 3 DAMTP, University of Cambridge, Wilberforce Road, Cambridge, CB3 0WA UK

## Abstract

We compute the total cross-section for *Z* boson production in bottom-quark fusion, applying to this case the method we previously used for Higgs production in bottom fusion. Namely, we match, through the FONLL procedure, the next-to-next-to-leading-log five-flavor scheme result, in which the *b* quark is treated as a massless parton, with the next-to-leading-order $\mathcal{O}(\alpha _s^3)$ four-flavor scheme computation in which bottom is treated as a massive final-state particle. Also, we add to our formalism the possibility of varying the heavy quark matching scale. The results obtained with the FONLL formalism can thus be compared directly to recent results obtained in various approximations, and used as a proxy to assess and discuss the issues of scale dependence and treatment of heavy quarks. Finally, We use our results in order to improve the prediction for the total *Z* production cross-section.

The production of a *Z* boson is one of the main standard candles at the LHC, and is now measured at the sub-percent level. The main production mode is through quark-anti-quark fusion, of which the bottom-initiated contribution accounts to $\mathcal{O}(4\%)$ of the total cross-section. This is a small but non negligible fraction of the total cross-section, and its contribution affects both the normalization and the shape of the kinematic distributions. Therefore a precise estimate of the bottom-initiated contribution is important for precision physics, for example in the determination of the *W* mass [1]. This process is thus an ideal test case for matched computations, recently applied to Higgs production in bottom quark fusion [2–5]. As we shall show here, it provides a theoretically transparent setting for the discussion of issues of choice of scheme and scale in the treatment of heavy quark contributions.

Like any process involving bottom quarks at the matrix-element level, the bottom-initiated *Z* production process may be computed using two different factorization schemes, which we refer to, as usual, as four- and five-flavour schemes for short. In the four-flavour scheme (4FS), the *b* quark is treated as a massive object, which decouples from QCD perturbative evolution. Calculations in this scheme are thus performed by only including the four lightest flavour together with the gluon in evolution equations for parton distributions (PDFs), and in the running of $\alpha _s$, so $n_f=4$ in the QCD $\beta$ function. In the five-flavour scheme (5FS), instead, the *b*  quark is treated on the same footing as other light quark flavors, there is a *b* PDF, and $n_f=5$ in evolution equations for PDFs and in the QCD $\beta$ function.

In matched calculations, both scheme are combined, in such a way that the result differs by that of each of the two schemes by terms which are sub-leading with respect to the accuracy of either of them. The FONLL scheme, first proposed for heavy quark production in hadronic collisions [6] has the advantage of being universally applicable; also, it allows for the matching of four- and five-flavour computations performed at any combination of individual perturbative orders. It has been extended to deep-inelastic scattering in Ref. [7] (also including [8, 9] the case in which the heavy quark PDF is independently parametrized) and, as mentioned, it has been used in Refs. [2, 3] for the computation of the total cross-section for Higgs production in bottom quark fusion.

**Fig. 1:**
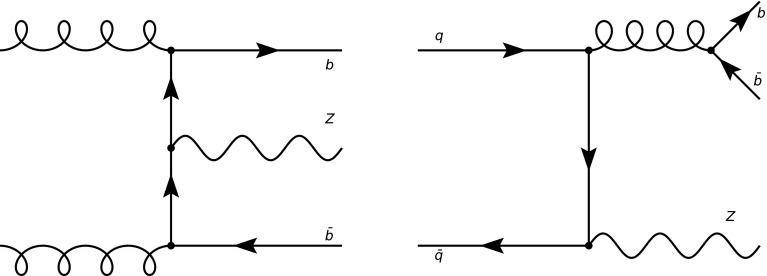
Leading-order diagrams for $Zb\bar{b}$ production in the four-flavor scheme: bottom fusion (left) and production from the initial state light quark (right)

Here, the methodology of Refs. [2, 3] is applied to *Z* production. When comparing Higgs to *Z* production in bottom quark fusion some care must be taken in defining exactly which process is being considered. Indeed, in the case of Higgs production the bottom fusion cross-section can be equivalently viewed as the cross-section for associate production in conjunction with a pair of *b* jets, i.e. as the $H b\bar{b}$ cross-section. In the case of *Z* production in the four-flavor scheme, on top of the leading-order *Z* production diagram in bottom fusion there is also a process in the quark-antiquark channel which produces the same $Z b\bar{b}$ final state, but in which the *Z* is radiated by initial-state light quarks and there are no *b* quarks in the initial state (see Fig. 1).

Hence, for *Z* production, unlike Higgs production, one may consider, at least in principle, two distinct processes. The first is *Z* production in bottom fusion, defined as *Z* production in the case in which the coupling of the *Z* to all quarks but bottom vanishes. In this case, only the diagram shown on the left of Fig. 1 contributes (as for Higgs production). The second is $Zb\bar{b}$ production, defined as the process with a *Z* and a bottom quark-antiquark pair in the final state, in which case both diagrams in Fig. 1 contribute. In the sequel we will consistently refer to the first definition (the one which is similar to Higgs) as *Z* production in bottom fusion, and to the second (the definition based on the final state) as $Zb\bar{b}$ production.

The possibility of separating experimentally the light quark- and gluon-initiated contributions of Fig. 1 to $Zb\bar{b}$ production has been discussed in Ref. [10], where it was shown that by choosing suitable kinematic variables it is possible to select regions in which the light quark contribution is dominant. However, from a theoretical point of view, the $Zb\bar{b}$ process is problematic because it is not infrared and collinear safe if the bottom mass is neglected, and thus it is beset by mass singularities in the 5FS. This is due to the fact that diagrams in which the bottom quark appears in the final state are counted as contributions to the $Zb\bar{b}$ process, but virtual corrections in which the *b* quark circulates in loops but is absent from the final state are not, and thus the cancellation of infrared singularities is incomplete. In the 4FS this leads to mass-singular contributions which are finite, but enhanced by double logs of the heavy quark mass. The problem is completely analogous to one which arises when defining heavy-quark deep inelastic structure functions, and was discussed in that context in Ref. [7], to which the reader is referred for a discussion of the way these double logs can be resummed.

Here, we will first focus on the construction of a matched computation of the process of *Z* production in bottom fusion, closely following the related case of Higgs production of Refs. [2, 3]. This will provide us with an interesting case study for issues related to scale choice and the relevance of matched computations. We will then turn to the use of this result as a means to improve the total *Z* cross-section, and in particular revisit the issue of the appropriate inclusion of light-quark initiated contributions to the $Zb\bar{b}$ process.

In the 5FS, the *Z*-production cross-section has been known up to next-to-next-to leading order (NNLO) (i.e. $\mathcal{O}(\alpha _s^2)$) for almost three decades [11] and the heavy-quark initiated contribution has been specifically discussed in several papers [12–14]. The next-to-leading order (NLO) ($\mathcal{O}(\alpha _s^3)$) four-flavour scheme $Zb\bar{b}$ production cross-section was originally computed in Ref. [15] for exclusive 2-jet final states, neglecting the *b*-quark mass. The *b*-quark mass was subsequently fully included in Refs. [16, 17].

Our first task is thus to use these two results in order to produce a matched computation for *Z* production in bottom fusion, following the procedure we presented in Refs. [2, 3] for the closely related case of Higgs production. Indeed, the counting of perturbative orders for these two processes is the same, and many of the Feynman diagrams are identical, with the only replacement of Higgs Yukawa couplings with gauge couplings. Following the nomenclature introduced in Refs. [2, 3] (and originally in Ref. [7] for DIS) we have constructed an FONLL-A result, which combines the NNLO 5FS with the LO $\mathcal{O}(\alpha _s^2)$ 4FS fully massive computation, and an FONLL-B, where instead the NNLO 5FS is matched to the full NLO $\mathcal{O}(\alpha _s^3)$ massive results.

Our construction is essentially identical to that of Refs. [2, 3], to which we refer for details: it can be obtained from it by simply replacing the matrix elements for Higgs production with those for gauge boson production. Specifically, we have computed the 5FS NNLO cross-section using the code of Ref. [14], which we cross-checked both at LO and NLO against MG5_aMC@NLO [18]. For the massive 4FS LO and NLO we have also used MG5_aMC@NLO. The construction of the FONLL matched results requires the computation of the massless limit of the massive result: we have implemented this in the public code [19] used in [3], in an updated version soon to be made public. All predictions are obtained using the NNLO NNPDF3.1 PDF set [20]. Lastly, PDF with varied thresholds are obtained using the APFEL evolution library [21]. In order to be consistent with the PDF set used we take, in the 4F scheme, the *b* pole mass to be $m_b=4.92$ GeV, while the strong coupling is run at NNLO, with $\alpha _s(m_Z) = 0.118$.

A new feature in comparison to Refs. [2, 3], is that we have now extended the formalism to allow for variation of the scale $\mu _b$ at which the 4FS and 5FS schemes are matched. In contrast, in previous FONLL implementations, scale was fixed at the bottom mass: $\mu _b=m_b$. The reason why results depend on a matching scale is that in the 5FS the *b* PDF is not independently parametrized. Rather, it is assumed that it is radiatively generated by the gluon. The matching scale is then the scale at which the *b* PDF is determined from the gluon. The interest in this is twofold. First, it allows us to perform a direct comparison with recent work [22], in which the impact of varying the matching scale is studied in the 5FS, and in particular it is argued that it might be advantageous to choose a very large value $\mu _b\gg m_b$. Second, studying the effect of matching scale variation provides us with another handle on the relative size of various contributions to the matched calculation, which we will study explicitly.

The matching condition itself depends on the matching scale in such a way that, at any given order, results are independent of it up to sub-leading corrections. This dependence persists in the FONLL matched results, but it is alleviated if the scale of the process is not too far from the bottom production threshold, because then the FONLL results almost reduces to the exact mass-dependent result in which the physical threshold is implemented exactly (as shown explicitly e.g. in Ref. [7]). It reappears when the scale of the process is high enough, in which case the FONLL result reduces to the 5FS, and it only goes away when computing the matching condition to increasingly high perturbative order, or by independently parametrizing the heavy quark PDF (indeed, this is the main motivation for independently parametrizing charm [20, 23]).

The generalization of the FONLL matching formulae of Refs. [2, 3] for a generic choice of matching scale is given in the Appendix. Dependence on this matching scale for Higgs in bottom fusion was studied explicitly in Refs. [4, 5]. The matching scheme of Refs. [4, 5], based on an EFT approach, was benchmarked in Ref. [24] to that of Refs. [2, 3] and found to agree with it at the percent level, hence a very similar dependence is expected for FONLL.

Our results are summarized in Figs. 2, 3, 4, where matched results in the FONLL-A and FONLL-B scheme are compared to each other and to the 4FS and 5FS scheme computations. Here and in the sequel all results are given for LHC at 13 TeV. Also, in Table 1, results for the cross-section in the three schemes with two different choices of central scale are collected, with uncertainties obtained from standard seven-point renormalization and factorization scale variation.

In the three plots we study respectively the renormalization, factorization, and matching scale dependence of the results. In each case, renormalization and factorization scales are fixed, and then varied about, either a high value $\mu =m_Z$, or a low value $\mu _{R,F}=\frac{m_Z+2m_b}{3}$. While the higher scale choice is standard in inclusive *W* and *Z* production, the lower choice was advocated in Refs. [25, 26] based on arguments that it is closer to the physical hard scale of the process, which corresponds to the average transverse momentum of the emitted partons, and leads to faster perturbative convergence. With this scale choice clearly the 4FS and 5FS are generally in better agreement.

Because we extend the plots down to very low values of the renormalization and factorization scales, for the preferred and most accurate FONLL-B case, we also provide an estimate of the ambiguity of the scale-varied result, in order to be able to assess whether and when the whole procedure becomes unreliable. This is done by performing scale variation in two different ways which differ by sub-leading terms. The two possibilities correspond to the observation (see e.g. [27]) that scale variation by a factor *k* of a quantity $F(\mu )$ which is scale-independent up to NLO but has a NNLO scale dependence can be performed by either letting

1 $$\begin{aligned} F(\mu _0;k)=F(\mu _0+\ln k)- \ln k \frac{d}{d\ln \mu } F(\mu )\Big |_{\mu _0=\mu _0+\ln k}, \end{aligned}$$

or

2 $$\begin{aligned} F(\mu _0;k)=F(\mu _0+\ln k)- \ln k \frac{d}{d\ln \mu } F(\mu )\Big |_{\mu =\mu _0}, \end{aligned}$$

where the first term on the r.h.s. is computed up to NLO, while the second term may be computed up to LO, and thus the two expressions differ by NNLO terms (and similarly for higher orders). The two options Eqs. (1–2) essentially correspond to changing the sign of the scale-variation terms, i.e., they amount to symmetrizing the scale variation: therefore, they are taken as the two extremes of a band which provides an estimate of the uncertainty on the scale uncertainty itself. Finally, matching scale variation is performed by varying it between the default $\mu _b=m_b$ and $\mu _b=2m_b$. When $\mu _b=2m_b$, only results for the choice Eq. 1 of scale variation are shown, but we have checked that the effect of varying the matching scale when renormalization and factorization scales are varied according to Eqs. 2 is similar, i.e., the whole uncertainty band moves up and down when $\mu _b$ is varied without changing shape significantly.

**Fig. 2:**
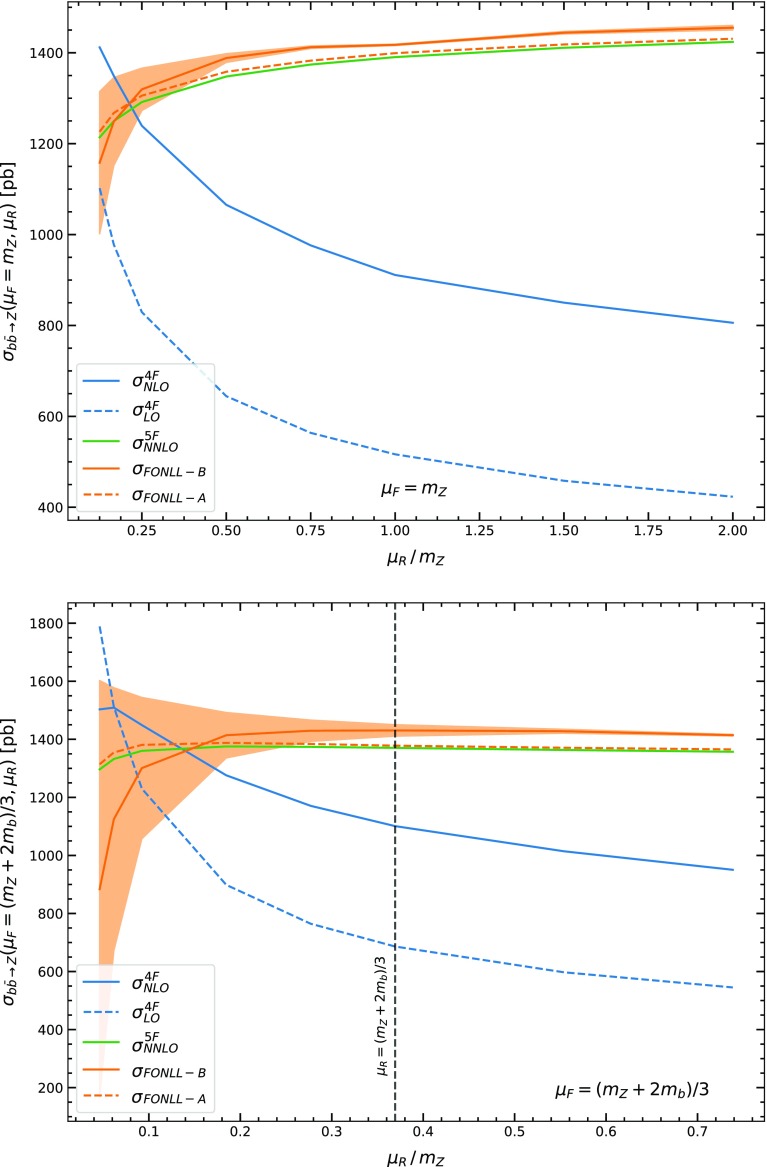
Comparison of the FONLL-A and FONLL-B matched results to each other, to the 4FS LO ($\mathcal{O}(\alpha _s^2)$) and NLO ($\mathcal{O}(\alpha _s^3)$), and to the 5FS NNLO. Results are shown as a function of the renormalization scale, with the factorization scale fixed at a high value $\mu _F=m_Z$ (top) or a low value $\mu _F=\frac{(m_Z+2m_b)}{3}$ (bottom). The band about the FONLL-B result is obtained from two different implementations of NLO scale variation that differ by NNLO terms (see text) and is thus an estimate on the ambiguity of the scale variation itself

**Fig. 3:**
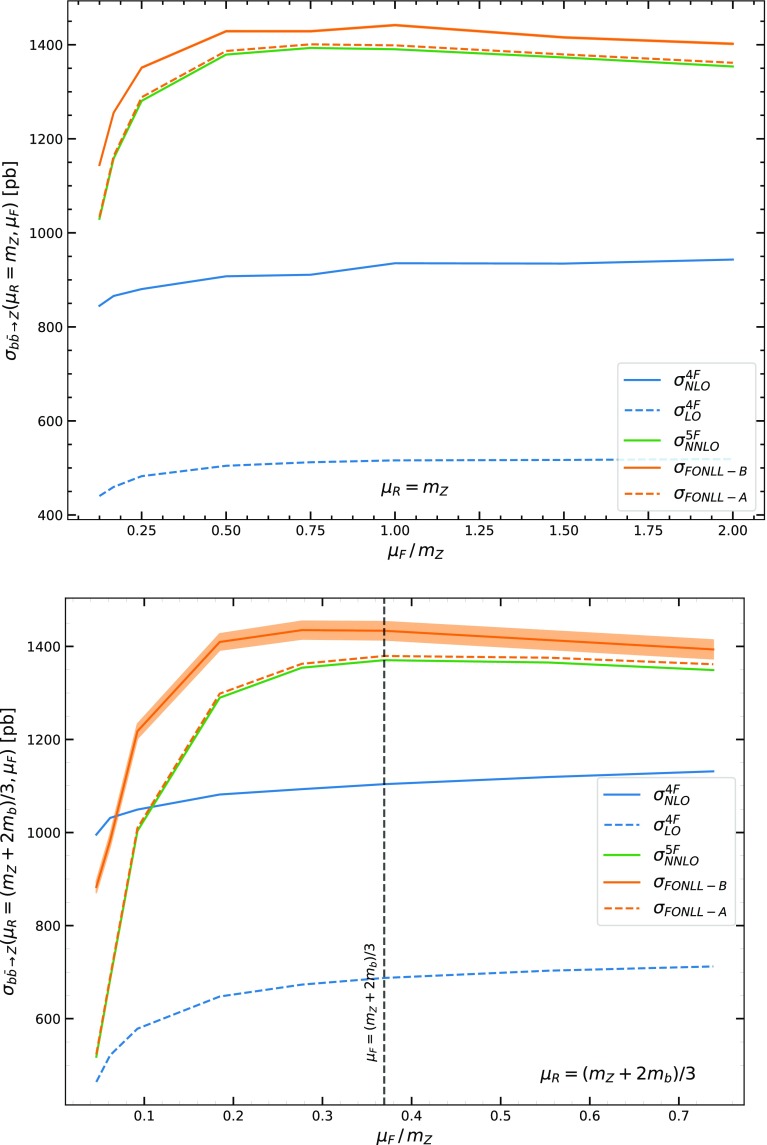
Same as Fig. 2, but now with the factorization scale varied with the renormalization scale kept fixed at a high value $\mu _R=m_Z$ (top) or a low value $\mu _R=\frac{(m_Z+2m_b)}{3}$ (bottom)

**Fig. 4:**
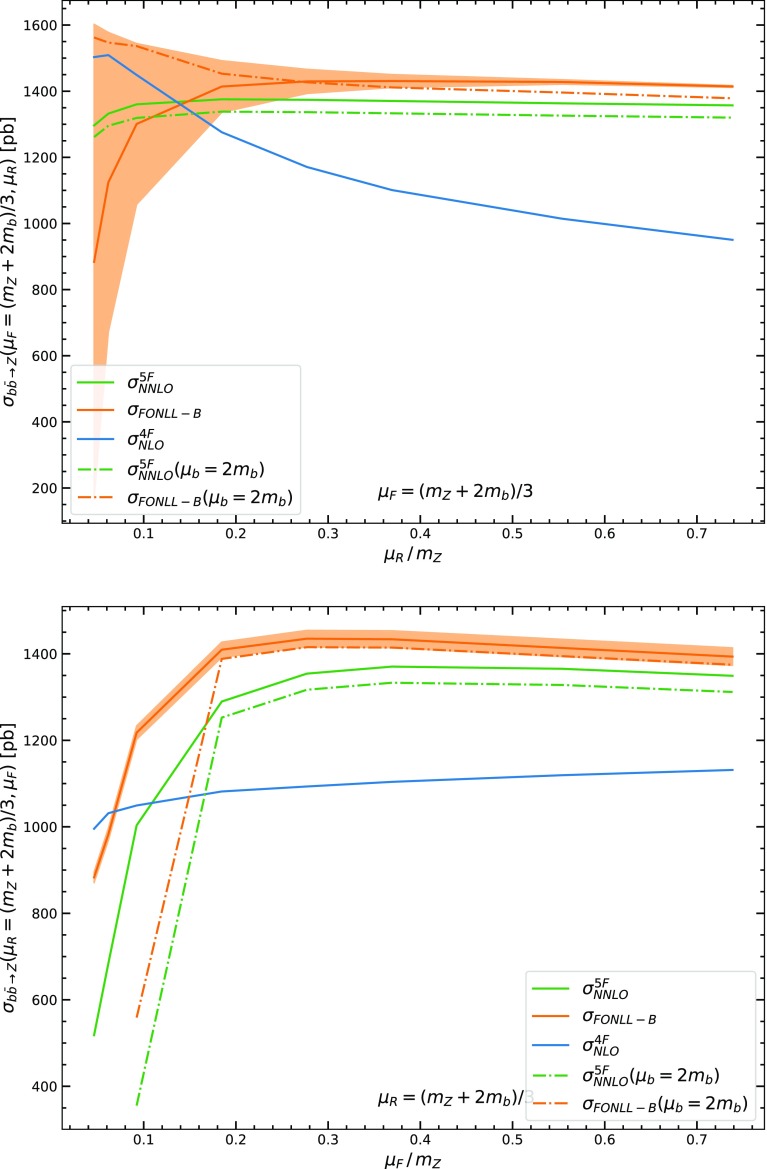
Comparison of the FONLL-B and of the 5FS NNLO results for two different values of the matching scale $\mu _b=m_b$ (same as in Figs. 2, 3) to each other and to the 4FS NLO. Results are shown as a function of the renormalization scale for fixed factorization scale (top) or as a function of the factorization scale for fixed renormalization scale (bottom), in each case with the fixed scale chosen as $\mu =\frac{(m_Z+2m_b)}{3}$. For the case $\mu _b=2m_b$ only the upper edge of the uncertainty band is shown

We first describe and comment our results, then discuss their interpretation, also in view of various approximations which have been suggested in the literature. A first observation is that comparison of Figs. 2, 3 to the corresponding plots for Higgs production in bottom fusion (Figs. 2–3 of Ref. [3]) show that they are qualitatively almost indistinguishable: this is not unexpected given the similarity between Higgs and *Z* production which we already repeatedly emphasized.

Coming now to these qualitative features we note that:

- The factorization scale dependence is generally very slight, while the renormalization scale dependence is, instead, stronger.
- The scale dependence is quite large in the 4FS scheme, even at NLO though it is reduced in comparison to the LO case. It is much weaker in the 5FS and FONLL cases which all have similar and similarly weak scale dependence, except for very low values $\mu _R\sim \frac{m_Z}{10}$ where however the ambiguity on the scale uncertainty blows up.
- The perturbative expansion is very unstable in the 4FS, with the LO and NLO results differing by a factor two or more. This instability is completely removed when the 4FS is matched to the 5FS: indeed, the FONLL-A and FONLL-B are quite close to each other.
- The 4FS and 5FS results are quite far from each other, with the 4FS NLO significantly closer to the 5FS than the LO. The FONLL results are in turn quite close to the 5FS.
- The perturbative expansion is indeed more stable for a lower choice of factorization and renormalization scale. For very low scales $\mu \sim \frac{m_Z}{10}$ the 4FS and 5FS results become similar, but the scale dependence becomes very large: in fact, the width of the uncertainty band becomes as wide as the scale variation in comparison to central scale choice, meaning that the results become unreliable.
- A change of matching scale has essentially the same effect on the 5FS and the FONLL results, and it has the effect of moving both towards the 4FS, though by a moderate amount.

These qualitative features have a simple theoretical interpretation. To this purpose, note that the cross-section for this process contains collinear logarithms regulated by the heavy quark mass, i.e. powers of $\ln \frac{\mu _Z^2}{m_b^2}$, one at each perturbative order. These logs arise from a transverse momentum integration, whose the upper limit is the maximum value of the transverse momentum, i.e. the hard scale of the process, which is proportional to but not equal to $m_Z$, and the lower limit is the physical production threshold, which is proportional to but not equal to $m_b$. Of course, one can always rewrite the ensuing logarithm as $\ln \frac{\mu _Z^2}{m_b^2}$, plus constants (i.e. terms which only depend on the dimensionless ratio $\tau =\frac{m_Z^2}{s}$), and mass corrections (i.e. terms suppressed by powers of $\frac{\mu ^2_b}{m_Z^2}$).

In the 4FS the result is exact, so whatever is not included in the log is included in the constants or in the mass corrections; on the other hand at N$^{k}$LO only the first $k+1$ logs are included. In the 5FS the logs are rewritten as $\ln \frac{\mu _Z^2}{m_b^2}=\ln \frac{\mu _Z^2}{\mu _F^2}+\ln \frac{\mu _F^2}{\mu _b^2}+\ln \frac{\mu _b^2}{m_b^2}$, where $\mu _F$ is the factorization scale and $\mu _b$ is the matching scale. The logs of the factorization scale $\ln \frac{\mu _F^2}{\mu _b^2}$ are then resummed to all orders into the evolution of the PDF, while the logs of the hard scale $\ln \frac{\mu _Z^2}{\mu _F^2}$ are included to finite order in the hard partonic cross-section and logs the matching scale, $\ln \frac{\mu _b^2}{m_b^2}$, are included to finite order in the matching condition, which expresses the initial *b* PDF in terms of the gluon (they would be implicitly included in the initial PDF if the *b* PDF were independently parametrized). When varying the factorization scale, logs at the upper end of the evolution are reshuffled between the resummed PDF and the fixed-order but exact hard cross-section. When varying the matching scale, logs at the bottom end of the evolution are reshuffled between the resummed PDF and the fixed-order but exact hard matching condition.

**Table 1:** Summary of results for the bottom fusion cross-section. Percentage error are obtained as the envelope of a standard 7-point $\mu _R,\mu _F$ variation around the central value $\mu _R=\mu _F=\mu$

	$\scriptstyle \sigma ^{4F}_{\text {NLO}}$	$\scriptstyle \sigma ^{5F}_{\text {NNLO}}$	$\scriptstyle \sigma _{\text {FONLL-B}}$
$\scriptstyle \mu = m_Z$	$\scriptstyle 935.36^{+13.9\%}_{-13.8\%}$ pb	$\scriptstyle 1390.46^{+2.41\%}_{-3.07\%}$ pb	$\scriptstyle 1443.32^{+1.17\%}_{-3.14\%}$ pb
$\scriptstyle \mu = \frac{m_Z+2m_b}{3}$	$\scriptstyle 1103.95^{+15.6\%}_{-13.9\%}$ pb	$\scriptstyle 1370.44^{+0.86\%}_{-5.89\%}$ pb	$\scriptstyle 1453.35^{+8.43\%}_{-2.71\%}$ pb

Note that both the hard coefficient and the matching condition contains logs and constants, but not mass-suppressed terms: so in the 5FS constants and logs of the matching scale, as well as constants and logs in the hard coefficient, are treated exactly but to fixed order, while logs of the factorization scale are resummed to all orders, but not constants. When the 5FS and the 4FS are matched into FONLL, also mass-suppressed terms, on top of constants and logs of the matching scale, are treated exactly.

The fact that the 4FS is perturbatively unstable while the 5FS is not then is easily explained as a manifestation of the fact that the 4FS contains large logs which are resummed in the 5FS. This is confirmed by the fact that the large difference between the 4FS LO and NLO is of the same order of the scale variation of the LO: indeed the scale variation by construction captures the size of logarithmic contribution. So the sizable difference which persists between the 5FS and NLO 4FS results is explained as being due to the higher order (NNLO and beyond) logs which are missing in the 4FS NLO, their size being quantitatively estimated in  [26]. This is confirmed by the observation that the FONLL-A and FONLL-B include both the large log resummation, and the full constants and mass-suppressed terms, up to LO and NLO respectively. The difference between the FONLL-A and FONLL-B is thus the size of the constant and mass-suppressed contributions to the difference between the 4FS LO and NLO. This is seen to be much smaller than the total difference between 4FS LO and NLO, which must therefore be due to the log.

In order to further disentangle, within this small contribution, the constant from mass-suppressed term, one would have to vary the hard scale, i.e. the *Z* mass. This was done in Ref. [3] for Higgs production: variation of the Higgs mass left the difference between FONLL-A and FONLL-B essentially unchanged, thus showing that mass corrections are negligible and the bulk of the difference between FONLL-A and FONLL-B is due to a constant. Given the similarity between the two processes we expect the same to be the case here. Given the small size of this contribution the issue is largely academic anyway.

The qualitative form of the renormalization scale dependence of the 4FS result is also easy to understand: as the scale is decreased, the value of $\alpha _s$ multiplying the large collinear log increases, and both the LO and NLO predictions grow; this growth is only partly reduced by the higher-order compensating term, at least down to scales $\mu _R\sim 0.2\mu _Z$ where the ambiguity on the scale variation itself becomes very large. The fact that the 5FS (and FONLL) result have almost no renormalization scale dependence shows that this scale dependence is coming from the *b* quark term which is treated differently between 4FS and 5FS.

The factorization scale dependence is particularly intriguing. The fact that this dependence is very slight in the 4FS is again consistent with the observation that scale dependence is driven by the heavy quark terms: in this scheme, in the absence of a *b* PDF, the factorization scale dependence is related to perturbative evolution of the light quarks and gluons, which is moderate at NNLO. On the other hand, in the 5FS (and in FONLL) collinear logs are resummed in the evolution of the *b*-PDF up to $\mu _F$, and then expanded out in the partonic cross-section from $\mu _F$ to the physical hard scale of the process. We therefore expect the factorization scale dependence in this scheme to be approximately stationary around this physical hard scale, very slight above it (where $\alpha _s$ is small) and to only become significant when $\mu _F$ is lower than the physical hard scale itself. This behaviour is clearly seen in Fig. 3, with the stationary point close to the low scale advocated in Refs. [25, 26] that indeed this scale of the hard process, and it nicely explains the very weak factorization scale dependence also seen in the 5FS unless $\mu _F\lesssim 0.2m_Z$ or so.

Finally, the fact that when increasing the matching scale $\mu _b$ the 5FS and FONLL-B result decrease and get closer to the 4FS is understood as a consequence of the fact that the higher-order resummed logs not included in the fixed order, $\ln \frac{\mu _F^2}{\mu _b^2}$, become smaller as $\mu _b$ increases. In the 4FS these logs are included at fixed order both in the hard matrix element and in the matching condition, in such a way that the dependence of $\mu _b$ cancels out to the given perturbative accuracy: the logarithmic contributiom and the constant that the 4FS result shares with the 5FS calculation remain the same as $\mu _b$ is changed. However, the remaining higher-order logarithms, which drive the difference bewteen the 4FS and the 5FS result become smaller as $\mu _b$ is raised.

We can finally discuss, in light of all this, the two related issues of choosing the various scales, $\mu _F$, $\mu _R$ and $\mu _b$, and of the validity of various approximations. As discussed, the scale dependence of this process is driven by the collinear logs in the *b*  quark contribution, and thus the bulk of it comes from the choice of argument in these logs.

In a fully massive 4FS calculation, these collinear logs are treated exactly, so the scale dependence comes purely from the choice of argument in the strong coupling. It then turns out that reducing the renormalization scale increases the 4FS unresummed results up to the point where it agrees with the 5FS resummed one. This is however accidental: the lack of resummation is made up by artificially increasing $\alpha _s$, and indeed at low scale the scale dependence of the 4FS result is not improved: if anything, it increases. Hence, the 4FS appears to be a poor approximation to this process and its improvement by lowering the renormalization scale is unreliable.

In a 5FS calculation, instead, as mentioned, the exact upper and lower limits of the transverse momentum integration are replaced by $\mu _F$ and $\mu _b$, respectively. As also mentioned, it has been argued [25, 26] that the exact, kinematics-dependent upper limit of integration is on average close to a scale $\frac{m_Z+2m_b}{3}\sim 0.35m_Z$. This is borne out by our results: for all $\mu _F\gtrsim 0.3m_Z$ the factorization scale dependence of the 5FS result is flat, and with this choice of $\mu _R$ the 5FS scale dependence is visibly flatter. Given the smallness of mass corrections, in practice a 5FS with low factorization and renormalization scales appears to be a good approximation of the full FONLL result.

On the other hand, it has been recently argued [22] that a higher choice of matching scale may provide a better approximation. Clearly, this is a process-dependent statement that should be checked on a case-by-case basis: as discussed raising the matching scale improves the accuracy of the starting, dynamically generated PDF, as it matches it at a scale where perturbation theory is more reliable, but it reduces the size of the logs which are resummed. In the present case, the resummed logs are a large effect and the constants a small correction, so raising the matching scale does not appear to be advantageous: indeed, the renormalization and factorization scale dependence is the same with $\mu _b=m_b$ or $\mu _b=2m_b$, with no obvious improvement.

**Fig. 5:**
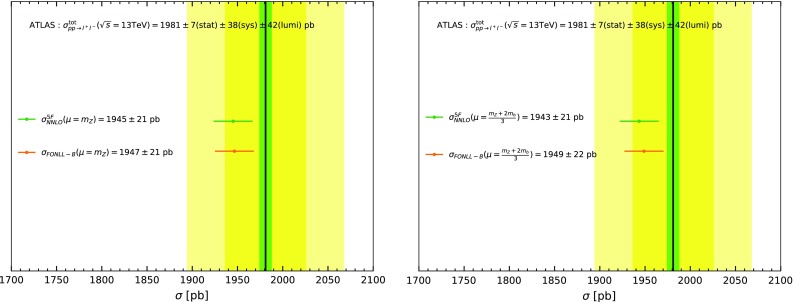
The total cross-section for *Z* production and decay into lepton pairs, computed in a pure five-flavour scheme, or with the bottom fusion contribution replaced by its FONLL-B expression. Results are show for two different choices of the renormalization and factorization scale for the bottom-induced contribution $\mu _R=\mu _F=m_Z$ (left) or $\mu _R=\mu _F=\frac{m_Z+2m_b}{3}$ (right); in both cases the remaining contributions to the total cross-sections are evaluated with $\mu _R=\mu _F=m_Z$. The uncertainty band is obtained from seven-point scale variation. Results are compared to the ATLAS measurement of Ref. [28]

In fact, when raising $\mu _b$ the 5FS result decreases, and moves towards the low 4FS, but with no improvement in perturbative stability of the latter. This is to be contrasted to the case in which $\mu _R$ and $\mu _F$ are lowered, which also brings the 4FS and the 5FS closer but now towards a high value, and with a visible increase in perturbative stability. In fact, the FONLL result shows that exact inclusion of the mass corrections (most likely the constant) increases the pure 5FS, by a small amount. On the contrary, raising the matching scale lowers it: this means that the deterioration of the log resummation is a larger effect than the improvement made by starting the PDF at a scale at which perturbation theory is more reliable. So a 5FS with large $\mu _b$ does not appear to be a better approximation in our case: it is likely to be a worse approximation if $\mu _b$ is raised by a moderate amount, and it definitely appears to be a poor approximation if $\mu _b$ is raised up to the point at which the 5FS result reduces to the 4FS one. On the other hand, a variation of $\mu _b$ by perhaps a factor two, as shown in Fig. 4, might well be a reasonable estimate of the uncertainty due to the use of a fixed-order matching condition and should be included in the theoretical uncertainty, as was done in Refs. [5, 24]. This theoretical uncertainty can only be removed by parametrizing the *b* PDF, in which case it is traded for a PDF uncertainty.

Having determined the total cross-section for *Z* production in bottom quark fusion at the highest available accuracy in a matched FONLL scheme, we can use this result in order to improve the total *Z* production cross-section. First, we recall that, as already mentioned, there are further contributions involving *b* quarks in the final state to the *Z* production cross-section, but without initial-state bottom in the 5FS, specifically at leading order the light-quark initiated contribution of Fig. 1. Bottom mass effects in these contributions could in principle also be included in a matched scheme. However, in order to perform the matching needed for a FONLL-B calculation ($\mathcal {O}(\alpha _s^3)$), one would need the $\mathcal {O}(\alpha _s^3)$ contributions to the light-quark-light-quark matching conditions, which are not available. Hence only an FONLL-A computation would be possible, instead of the more accurate FONLL-B.

Furthermore, also as already mentioned, a matched computation including these contributions must be performed at the level of the total *Z* cross-section, rather than that for the $Zb\bar{b}$ cross-section, because these real emission contribution are affected by infrared divergences which cancel against virtual correction in which the *b* quark circulates in loops but there are no *b* quarks in the final state.

However, we can estimate the size of these contributions and the impact of their FONLL improvement by computing the leading-order contribution Fig. 1 by removing the infrared divergence through an invariant mass cut $m_{b\bar{b}}\ge \sqrt{2} m_b$. We then get (with the low scale choice and all other settings of the previous calculation) a contribution $\sigma ^\mathrm{light}_\mathrm{5FS}(Zb\bar{b})=146.1$ pb from the diagram of Fig. 1 in the 5FS. The corresponding 4FS result is $\sigma ^\mathrm{light}_\mathrm{4FS}(Zb\bar{b})=129.5$ pb, while the massless limit of the 4FS result is $\sigma ^\mathrm{light}_\mathrm{0}(Zb\bar{b})=138.8$ pb. It follows that the effect of the FONLL-B improvement over a pure 5FS computation of this term is at the level of less than 1% of the total $b\bar{b}$ cross-section of Table 1, to be compared to the 5-6% impact of the FONLL-B improvement of the bottom fusion contribution seen in Table 1. Therefore, the FONLL improvement of the light-quark initiated contribution appears unnecessary.

We can thus consider the total cross-section. We compute this at NNLO in the 5FS, using the same code and settings discussed above, and then improve it by subtracting the bottom-initiated contribution to it, namely, the cross-section for *Z* production in bottom fusion, and replacing it with its FONLL expression as defined and discussed above. Because both the total and the bottom-fusion cross-section are separately collinear safe, this leads to a consistent result. Results are shown in Fig. 5 for the rate into lepton pairs, obtained multiplying by the branching ratio B(Z$\rightarrow$ll)$=3.3658~10^{-2}\pm 0.0023~10^{-2}$ [29]. The uncertainty shown is obtained from standard seven-point scale variation, with the central result given as the mid-point of the band. The bottom fusion contribution is computed with either of the choices of scale that we used, with the total cross-section determined with $\mu _R=\mu _F=m_Z$. At the level of total cross-section, the effect of the bottom mass is very minor, at the permille level, much smaller than the NNLO scale uncertainty. This justifies neglecting the further FONLL improvement of the light-quark induced bottom production contribution, which at the level of total cross-section would be possible, but, as we have seen above, would have a yet much smaller impact.

In summary, we have determined the total cross-section for *Z* production in bottom quark fusion at the highest available accuracy in a matched FONLL scheme, and we have used our results as a test case for the discussion of issues of scale dependence and heavy quark treatment, by generalizing our previous results for Higgs production, and studying not only renormalization and factorization scale, but also matching scale dependence. We have finally assessed the impact of the FONLL improvement both on the bottom fusion and total *Z* production cross-section.

Our main phenomenological conclusion is that, similarly to the case of Higgs production, mass effects on the bottom fusion cross-section are small, but non-negligible in comparison to the high experimental accuracy to which this process can be measured. However, their impact on the total *Z* cross-section is quite small, given that the bottom fusion contribution is only a small fraction of the total. For bottom fusion, the contribution due to the resummation of collinear logs of the heavy quark is sizable, thereby making a five-flavour scheme in which the *b* quark is endowed with a PDF a better approximation to the full FONLL result than the fixed-order 4FS calculation with massive b, which falls short of the full prediction and displays large scale uncertainties. A low choice of renormalization and factorization scale reduces the scale dependence of both the full FONLL and pure 5FS result and is likely to improve their accuracy, though in practice this makes little difference as the scale dependence of both these results is very slight. However, it does suggest that the hard physical scale for this process is lower than the final-state mass, as previously advocated.

All in all, our results support the conclusion that, when dealing with processes involving heavy quarks, a fully matched treatment of heavy quarks with a proper inclusion of mass effects is necessary for LHC phenomenology at the percent level, either through its direct use, or as a guide to construct efficient and accurate approximations.

A public implementation of our NNLL+NLO FONLL-B matched computation will be added to our code for Higgs production [3], publicly available from http://bbhfonll.hepforge.org/.

## A FONLL expressions with $\mu _b$ different from $m_b$

We give for completeness the FONLL expressions by using $m_b$ different from $\mu _b$. Note that the only difference with respect to the formulae presented in [3], is in the logarithm obtained from the expansion of the *b* PDF, where $L=\log (Q^2/m_b^2)$, becomes $L=\log (Q^2/\mu _b^2)$.

With this modification in place we get, for the 4F scheme, *B* coefficients:

A.1 $$\begin{aligned} B_{gg}^{(2)}\left( y,\frac{Q^2}{m^2_b}\right)&= \hat{\sigma }_{gg}^{(2)}\left( y,\frac{Q^2}{m_b^2}\right) \end{aligned}$$

A.2 $$\begin{aligned} B_{q\bar{q}}^{(2)}\left( y,\frac{Q^2}{m^2_b}\right)&= \hat{\sigma }_{q\bar{q}}^{(2)}\left( y,\frac{Q^2}{m_b^2}\right) \end{aligned}$$

while at $\mathcal{O}(\alpha _s^3)$ the redefinition of $\alpha _s$ contributes:

A.3 $$\begin{aligned}&B_{gg}^{(3)}\left( y,\frac{Q^2}{m^2_b},\frac{\mu _R^2}{\mu _b^2},\frac{\mu _F^2}{\mu _b^2}\right) = \hat{\sigma }_{gg}^{(3)}\left( y,\frac{Q^2}{m_b^2}\right) \nonumber \\&\qquad \qquad \qquad \qquad \qquad \qquad \qquad \;- \frac{2 T_R}{3\pi } \ln {\frac{\mu _R^2}{\mu _F^2}}\hat{\sigma }_{gg}^{(2)}\left( y,\frac{Q^2}{m_b^2}\right) \end{aligned}$$

A.4 $$\begin{aligned}&B_{q\bar{q}}^{(3)}\left( y,\frac{Q^2}{m^2_b},\frac{\mu _R^2}{\mu _b^2},\frac{\mu _F^2}{\mu _b^2}\right) = \hat{\sigma }_{q\bar{q}}^{(3)}\left( y,\frac{Q^2}{m_b^2}\right) \nonumber \\&\qquad \qquad \qquad \qquad \qquad \qquad \qquad \;- \frac{2 T_R}{3\pi } \ln {\frac{\mu _R^2}{\mu _b^2}}\hat{\sigma }_{q\bar{q}}^{(2)}\left( y,\frac{Q^2}{m_b^2}\right) \end{aligned}$$

A.5 $$\begin{aligned}&B_{gq}^{(3)}\left( y,\frac{Q^2}{m^2_b}\right) = \hat{\sigma }_{gq}^{(3)}\left( y,\frac{Q^2}{m_b^2}\right) \end{aligned}$$

A.6 $$\begin{aligned}&B_{qg}^{(3)}\left( y,\frac{Q^2}{m^2_b}\right) = \hat{\sigma }_{qg}^{(3)}\left( y,\frac{Q^2}{m_b^2}\right) . \end{aligned}$$

The massless limit of the 4F scheme coefficients, $B^{(0)}$, are, in this case, given by

A.7 $$\begin{aligned}&\sigma ^{(4),(0)}\left( \alpha _s(Q^2),L\right) \nonumber \\&\quad =\int _{\tau _H}^{1} \frac{dx}{x}\int _{\frac{\tau _H}{x}}^{1} \frac{dy}{y^2}\sum _{ij=q,g}f_{i}(x,Q^2)f_j\left( \frac{\tau _H}{x y},Q^2\right) \nonumber \\&\quad B_{ij}^{(0)}\left( y,L,\alpha _s(Q^2)\right) , \end{aligned}$$

with

A.8 $$\begin{aligned} B_{ij}^{(0)}\left( y,L,\alpha _s(Q^2)\right) = \sum _{p=2}^N\left( \alpha _s(Q^2)\right) ^pB_{ij}^{(0),(p)}\left( y,L\right) , \end{aligned}$$

and

A.9 $$\begin{aligned}&B_{gg}^{(0)(2)} (y,L) = y\int _y^1\frac{dz}{z}\left[ 2\mathcal {A}_{gb}^{(1)}\left( z,L\right) \mathcal {A}_{gb}^{(1)}\left( \frac{y}{z},L\right) \nonumber \right. \\&\qquad \qquad \qquad \qquad \left. + 4\mathcal {A}_{gb}^{(1)}\left( \frac{y}{z},L\right) \hat{\sigma }_{gb}^{(1)}(z)\right] + \hat{\sigma }_{gg}^{(2)}(y), \end{aligned}$$

A.10 $$\begin{aligned}&B_{q\bar{q}}^{(0)(2)} (y,L) = \hat{\sigma }_{q\bar{q}}^{(2)}(y); \end{aligned}$$

while the new contributions to $\mathcal{O}(\alpha _s^3)$ are

A.11 $$\begin{aligned} B_{gg}^{(0)(3)} (y,L)&= y\int _y^1\frac{dz}{z}\left[ 4\mathcal {A}_{gb}^{(2)}\left( z,L\right) \mathcal {A}_{gb}^{(1)}\left( \frac{y}{z},L\right) \nonumber \right. \\&\quad \left. + 2\mathcal {A}_{gb}^{(1)}\left( z,L\right) \mathcal {A}_{gb}^{(2)}\left( \frac{y}{z},L\right) \hat{\sigma }_{b\bar{b}}^{(1)}(z) \right. \nonumber \\&\quad \left. + 4\mathcal {A}_{gb}^{(2)}\left( \frac{y}{z},L\right) \hat{\sigma }_{gb}^{(1)}(z)\nonumber \right. \\&\quad \left. + 4\mathcal {A}_{gb}^{(1)}\left( \frac{y}{z},L\right) \hat{\sigma }_{gb}^{(2)}(z)\right] , \end{aligned}$$

A.12 $$\begin{aligned} B_{gq}^{(0)(3)} (y,L)&= y\int _y^1\frac{dz}{z}\left[ 2\mathcal {A}_{\Sigma b}^{(2)}\left( z,L\right) \mathcal {A}_{gb}^{(1)}\left( \frac{y}{z},L\right) \nonumber \right. \\&\left. \quad + 2\mathcal {A}_{\Sigma b}^{(2)}\left( \frac{y}{z},L\right) \hat{\sigma }_{gb}^{(1)}(z) \right. \nonumber \\&\quad \left. + 2\mathcal {A}_{gb}^{(1)}\left( \frac{y}{z},L\right) \hat{\sigma }_{qb}^{(2)}(z)\right] , \end{aligned}$$

which completes our result in the case in which $\mu _b\ne m_b$.
